# Development of In Vitro Potency Methods to Replace In Vivo Tests for Enterovirus 71 Inactivated Vaccine (Human Diploid Cell-Based/Vero Cell-Based)

**DOI:** 10.3390/vaccines13040404

**Published:** 2025-04-13

**Authors:** Xuanxuan Zhang, Li Yi, Dan Yu, Jun Li, Xintian Li, Xing Wu, Fan Gao, Qian He, Wenhui Wang, Kaiwen Wang, Zejun Wang, Zhengling Liu, Yadong Li, Yong Zhao, Huiyi Li, Xiao Ma, Qingbing Zheng, Longfa Xu, Tong Cheng, Rui Zhu, Jing Guo, Jing Li, Qunying Mao, Zhenglun Liang

**Affiliations:** 1 NHC Key Laboratory of Research on Quality and Standardization of Biotech Products, NMPA Key Laboratory for Quality Research and Evaluation of Biological Products, State Key Laboratory of Drug Regulatory Science, Research Units of Innovative Vaccine Quality Evaluation and Standardization, Chinese Academy of Medical Sciences, National Institutes for Food and Drug Control, Beijing 102629, China; zhangxx_01@163.com (X.Z.); eastarwx@163.com (X.W.); gaofan@nifdc.org.cn (F.G.); hq5740@126.com (Q.H.); maxiao@nifdc.org.cn (X.M.); lzhenglun@126.com (Z.L.); 2 Institute of Medical Biology, Chinese Academy of Medical Sciences, Kunming 650118, China; yl@imbcams.com.cn (L.Y.); liuzhengling@imbcams.com.cn (Z.L.); yadong_li@imbcams.com.cn (Y.L.); zhaoyong@imbcams.com.cn (Y.Z.); 3 Sinovac Biotech, Beijing 100085, China; yud@sinovac.com (D.Y.); lijun@sinovac.com (J.L.); lixt8378@sinovac.com (X.L.); lij@sinovac.com (J.L.); 4 Wuhan Institute of Biological Products Co., Ltd., Wuhan 430207, China; wangwenhui583@163.com (W.W.); kevin30596@163.com (K.W.); wangzejun@sinopharm.com (Z.W.); guojing27@sinopharm.com (J.G.); 5 Key Laboratory of Research on Quality and Standardization of Biotech Products, Chinese Pharmacopoeia Commission, Beijing 100061, China; lihuiyi@chp.org.cn; 6 State Key Laboratory of Vaccines for Infectious Diseases, Xiang An Biomedicine Laboratory, School of Public Health, Xiamen University, Xiamen 361005, China; abing0811@xmu.edu.cn (Q.Z.); longfaxu@xmu.edu.cn (L.X.); tcheng@xmu.edu.cn (T.C.)

**Keywords:** enterovirus 71 inactivated vaccine, in vitro relative potency, in vivo potency, correlation, replacement

## Abstract

Background: The three commercial Enterovirus 71 (EV71) inactivated vaccines which have effectively controlled the EV71 pandemic currently rely on inherent variable in vivo potency methods for batch release. To align with 3R (Replacement, Reduction, Refinement) principles and enhance quality control, this study referred to WHO guidelines and the European Pharmacopoeia to develop in vitro relative potency (IVRP) methods. Methods: Working standards tracing to phase 3 clinical vaccines were established. Manufacture-specific IVRP methods were developed and validated per ICH Q14/Q2(R2), utilizing conformational epitope-targeting neutralizing monoclonal antibodies (MAbs). One of the MAbs (CT11F9) recognition sites was clarified with Cryo-EM. Subsequently, the performance of IVRP was assessed using varied concentrations and heat-treated vaccines. The correlation between IVRP and in vivo methods was analyzed, followed by setting IVRP specifications. Results: The manufacturer-specific working standard exhibited ED50 values comparable to those of related phase 3 clinical vaccines. All IVRP methods achieved a relative bias/precision/total error ≤ 15%. The IVRP methods correlated with in vivo methods (*p* < 0.05, r > 0.9) can discriminate EV71 antigen concentrations (*p* < 0.01, r > 0.99) and indicate the stability of the vaccines. Cryo-EM was adopted to identify the epitopes recognized by CT11F9, revealing that this neutralizing antibody recognizes a conformational epitope spanning VP1-3 of the same protomer. Using 31–47 batches of commercial vaccines, IVRP specifications were proposed as 0.56–1.35, 0.58–1.40, and 0.54–1.50. Conclusions: Based on conformational epitope-targeting neutralizing MAbs, manufacturer-specific IVRP methods, which were sensitive to process variations and correlated with in vivo results, have been established. IVRP methods provide a reliable, animal-free alternative for EV71 vaccine batch release.

## 1. Introduction

Enterovirus 71 (EV71) is a major pathogen causing severe hand, foot, and mouth disease (HFMD) in children and infants, threatening public health in the Western Pacific Region [1]. In 2009, mainland China reported over 1.15 million HFMD cases and 353 deaths [1]. To tackle this outbreak, supported by Chinese national science and technology plans, the National Institutes for Food and Drug Control (NIFDC) and three manufacturers formed a team for the EV71 inactivated vaccine development. Two companies used Vero cells and one used human diploid cells to produce EV71 inactivated vaccines through inactivation, purification, and aluminum adjuvant adsorption processes [2,3]. Since their launch in 2015–2016, the three vaccines have shown good safety and efficacy, with an efficacy of over 90% against EV71-related diseases in mainland China [4,5,6]. The vaccines have been consistently produced for 9 years, with mature processes and stable quality. Currently, the in vivo assay is used for batch potency testing of EV71 inactivated vaccines, requiring mice with a 28-day testing period.

In vitro potency methods, in line with the 3R principles, offer advantages in precision and shorter testing periods. The WHO and national regulators encourage replacement research for in vivo batch testing [7,8,9]. However, only a few vaccines, such as inactivated hepatitis A, recombinant hepatitis B, and conjugated polysaccharide vaccines, have already undergone the transition from in vivo methods to in vitro methods for quality control [10,11,12]. Replacement research for vaccines like the rabies vaccine, tick-borne encephalitis vaccine, diphtheria vaccine, tetanus vaccine, and acellular pertussis vaccine is still ongoing [13,14,15,16,17]. This is due to challenges in vaccine potency method replacement, such as difficulties in determining the correlation between in vivo and in vitro methods because of the high variability of in vivo methods, complications in regulatory approval due to changes in specification caused by in vitro methods, and issues of consistency and compliance in method replacement research for vaccines produced by multiple companies with different processes.

Supported by a Chinese Pharmacopoeia Commission project, the NIFDC and three EV71 inactivated vaccine manufacturers conducted research on replacing the in vivo potency method with an in vitro one for EV71 inactivated vaccine batch testing based on WHO guidelines, European Pharmacopoeia method replacement guidelines, ICH Q2 (R2)/Q14, and the relevant general chapter of the Chinese Pharmacopoeia (2025 edition) [7,9,18,19,20]. Each company established working standards consistent with the immunogenicity of their phase 3 clinical trials, developed in vitro relative potency (IVRP) methods meeting preset analytical target profile (ATP) requirements, and established specifications for their vaccines.

## 2. Materials and Methods

### 2.1. Working Standard Development and Calibration

Each of the three companies selected a vaccine batch with immunogenicity similar to phase 3 clinical vaccines as the working standard. In line with the WHO manual and the Chinese Pharmacopoeia Volume III (2020 edition) [21,22], they used in-house EV71 antigen detection methods, with the national standard for enterovirus 71 (EV71) antigen content (300016-202002, 2320 IU/mL) as the reference, to conduct multiple independent tests on the working standard. Minitab 19 software was used for the Anderson–Darling normality testing of the independent test data; *p* > 0.05 indicated normal distribution. The results showed no abnormal or outlier values for antigen content calibration. The mean and 95% confidence interval (CI) of the test data were calculated to obtain the calibration value.

### 2.2. Development and Validation of the IVRP Method

#### 2.2.1. ATP Setting

To establish a rapid and accurate EV71 IVRP method, ICH Q14 and Q2 (R2) and prior knowledge were referred to set the acceptable standards for specificity, precision, and accuracy [18,19].

#### 2.2.2. Risk Assessment

Various factors affecting ELISA were visualized in an Ishikawa diagram (Figure S1). The factors were divided into six parts: analyst, equipment, method parameters, environment, calculation, materials, and reagents. Risk assessment was carried out based on each factor’s impact on the ATP’s accuracy, precision, and specificity.

#### 2.2.3. Optimization of ELISA Method

A fixed-effect or design of experiment (DoE) was used to optimize the identified critical factors. For the DoE design, JMP 13’s custom design was used to generate an experimental plan table.

#### 2.2.4. Model Selection

Detection results of serially diluted working standards were fitted using common models (e.g., four-parameter logistic Rodbard, Linear model). The fitting method’s AICc, growth rate, and R^2^ were analyzed to choose the appropriate one.

#### 2.2.5. Specificity Validation

Each company used the newly established ELISA method to detect matrix solutions (e.g., cell growth medium, virus dilution buffer) and other inactivated vaccine bulk liquids produced by the manufacturer.

#### 2.2.6. Precision and Accuracy Validation of IVRP

According to USP 1033 [23], vaccines with relative potency levels of 0.5–2.0 were repeatedly tested at least 16 times by different analysts at different times. The results were used to evaluate the method’s precision and accuracy.

#### 2.2.7. Method Capability Evaluation Indicator of IVRP

BMV software (V1.0, NIFDC, Beijing, China) was used to analyze method capability. The total method error, prediction interval, tolerance interval, method capability indices, and method misjudgment probability of the relative potency levels 0.5–2.0 were comprehensively analyzed.

### 2.3. Epitope Identification of CT11F9 MAb

#### 2.3.1. Cryo-EM Sample Preparation and Data Collection

Immune complexes were prepared by incubating EV71 mature virions with CT11F9 Fab at a mass ratio of 1:1.2 at 37 °C for 60 min. Aliquots (3 μL) of the immune complexes were applied to freshly glow-discharged holey carbon Quantifoil Cu grids (R2/1, 200 mesh, Quantifoil Micro Tools, Großlöbichau, Germany). The grids were blotted for 5 s at 100% relative humidity and 4 °C before plunge-freezing in liquid ethane cooled by liquid nitrogen using a Vitrobot Mark IV (Thermo Fisher Scientific, Waltham, MA, USA). Cryo-EM datasets were acquired using K3 direct electron detectors mounted on a Tecnai F30 electron microscope (Thermo Fisher Scientific) at nominal magnifications of 31,000× *g*, corresponding to pixel sizes of 1.00 Å, respectively. Each movie was recorded with a total electron dose of approximately 40 e^−^/Å^2^ and fractionated into 41 frames with an exposure time of 3 s. Data collection was automated using SerialEM 3.8.5 software [24].

#### 2.3.2. Image Processing and Three-Dimensional Reconstruction

Movie frames were aligned and averaged using MotionCorr2 [25], and contrast transfer function (CTF) parameters were determined using Gctf [26]. Micrographs exhibiting excessive drift or astigmatism were excluded from further processing. Particles were automatically picked and screened using the cryoSPARC 2.4.3 [27]. Multiple rounds of reference-free 2D classification and 3D classification were performed using cryoSPARC 4.5.3 to select high-quality particles for final refinement. The selected particles were subjected to homogeneous refinement and post-processing using cryoSPARC 4.5.3. The final resolution was assessed using the gold-standard Fourier shell correlation (FSC) with a threshold of 0.143 [28].

#### 2.3.3. Model Building and Refinement

The cryo-EM structure of the EV71 mature virion (PDB code: 3VBS) was used as a homology model for the EV71-based immune complexes. The initial atomic model for the variable domain of the CT11F9 Fab fragment was generated by homology modeling using Accelrys Discovery Studio 2017 R2 software. The initial model was fitted into the segmented cryo-EM density map (including an asymmetric unit) using UCSF Chimera [29] and manually rebuilt in Coot [30]. The model was further refined using phenix.real_space_refine in PHENIX [31]. After refinement, the model was fitted into the density of six neighboring protomers, and the entire complex was refined as a single unit to optimize steric clashes. Model statistics, including bond lengths, bond angles, all-atom clashes, rotamer distributions, and Ramachandran plot values, were carefully inspected in Coot throughout the refinement process. The final atomic model was validated using Molprobity [32]. Model statistics are summarized in Table S1. Buried surface areas of CT11F9 and intermolecular interactions were analyzed using the PISA server (https://www.ebi.ac.uk/pdbe/pisa/ (accessed on 25 December 2024)), with hydrogen bonds defined by donor-to-acceptor distances ≤ 4 Å. All structural figures were generated using UCSF ChimeraX [33].

### 2.4. In Vivo Potency Assay

Vaccine samples were serially diluted and intraperitoneally injected into 42–56-day-old or 18–22 g BALB/c mice (5 serial dilutions, 10 mice per dilution, 1.0 mL per mouse). Ten mice were used as controls. After 4 weeks, blood was collected, and serum EV71-neutralizing antibody titers were detected (titers ≥ 1:8 considered positive). ED50 was calculated using the Reed–Muench or Karber method.

### 2.5. Neutralizing Assays

MAbs (adjusted to 1 mg/mL) and mouse blood (diluted 8-fold and heated at 56 °C for 30 min) were serially diluted 2-fold for 8 dilutions. A total of 50 μL EV71 virus (FY7VP5, H07, FY23KB) at 100 CCID50/0.05 mL was added to sample wells and incubated at 37 °C for 2 h. Then, 1.0 × 10^4^–1.5 × 10^4^ RD cells (RD cells: ATCC (Manassas, VA, USA), CCL-136, a gift from the National Vaccine & Serum Institute, Beijing, China) were added per well and cultured in a 35 °C CO_2_ incubator for 7 days. The cytopathic effect (CPE) was observed with an inverted microscope, and the reciprocal of the highest dilution inhibiting 50% of the CPE was taken as the endpoint titer.

### 2.6. Western Blotting Analysis

EV71 purified antigen was mixed with loading buffer and boiled. The heated samples were electrophoresed in 10% SDS-PAGE and then transferred to polyvinylidene fluoride (PVDF) membranes. The membranes were incubated with 5% skim milk in phosphate-buffered saline with Tween 20 (PBST) at room temperature for 3 h and then cut into individual strips. One strip was incubated with a monoclonal antibody (MAb) specific for linear epitopes as a positive control. Other strips were incubated with MAbs from the three companies, followed by washing and incubation with HRP-anti-mouse (1:5000). Results were visualized with electrochemiluminescence (ECL) hypersensitive luminous solution and detected with a charge-coupled device imaging system.

### 2.7. Specification Establishment

Commercial batches of vaccines from 2020 to 2023 were selected for IVRP testing. GraphPad 9.0 was used to create individual control charts for the results of the finished EV71 vaccines. Based on the results and the “3SD rule”, specifications were proposed.

### 2.8. Statistical Analysis

GraphPad 9.0 was used to perform Spearman correlation analysis on the theoretical and measured values of IVRP and ED50. A *p* < 0.05 indicated a statistically significant difference between two variables. Correlation coefficient r: 0.8 < |r| < 1.0, very strong correlation; 0.6 < |r| < 0.8, strong correlation; 0.4 < |r| < 0.6, moderate correlation; 0.2 < |r| < 0.4, weak correlation; 0 < |r| < 0.2, very weak or no correlation.

## 3. Results

### 3.1. Establishment of IVRP Working Standards

Biological activity assay methods can vary greatly and are not suitable for absolute quantification. The FDA has proposed using relative potency values to reflect biological activity [23]. Therefore, establishing IVRP working standards is crucial for the IVRP method. In line with the WHO guidelines and the Chinese Pharmacopoeia, the NIFDC collaborated with three manufacturers (A, B, and C) to establish working standards [21,22]. Vaccines produced by the same process as clinical vaccines were used as working standards. The results showed that the ED50 value and antigen content of the working standards were comparable to those of the phase 3 vaccines (Table 1). To ensure traceability, each company used the national standard for EV71 antigen content (300016-202002, 2320 IU/mL) as the reference to calibrate working standards. All data acquired by the in-house method were analyzed for normality and homogeneity of variance, with no abnormal or outlier values. Statistical analysis showed that the antigen contents of the working standards prepared by manufacturers A, B, and C were 242 IU/mL (95% CI: 240–245 IU/mL), 812 IU/mL (95% CI: 793–830 IU/mL), and 999 IU/mL (95% CI: 985–1012 IU/mL), respectively (Table 1).

### 3.2. Establishment and Validation of an IVRP for EV71 Inactivated Vaccine Batch Testing

The three manufacturers developed IVRP methods for EV71 inactivated vaccine batch testing in accordance with the requirements of ICH Q2 (R2)/Q14 [18,19]. Here is a detailed introduction, taking Manufacturer A as an example.

#### 3.2.1. Establishment of an IVRP

##### Establishment of the ATP

The in vitro method was designed to replace the in vivo assay for EV71 inactivated vaccine batch and stability testing. Per the European Pharmacopoeia 5.2.14 guidelines, quality control methods of vaccines should reflect antigen content and function [7]. An ELISA based on neutralizing monoclonal antibodies (MAbs) targeting conformational epitopes is a promising approach. Referring to existing ELISA protocols and practical requirements, the ATP for the method was defined (Table S1). The method should have no cross-reactivity with vaccine matrix solutions, hepatitis A bulk, inactivated polio bulk, or coxsackievirus harvest. The accuracy (relative bias) should be within ±15%, with a 90% confidence interval of ±20%. The precision should be ≤15%, and the total analytical error should be ≤20%.

##### Research on MAb

**Figure 1:**
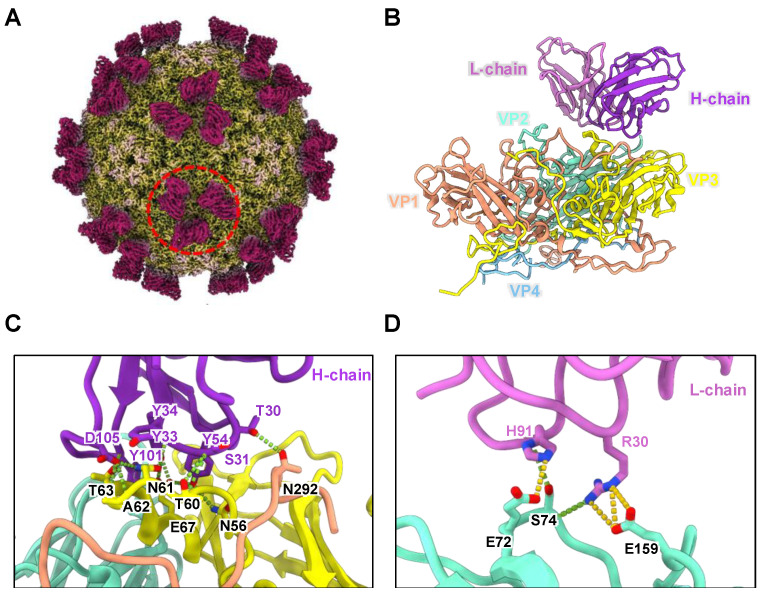
Structural density, atomic model, and interaction interface of the EV71:CT11F9 immune complex. (**A**) Structural density of the EV71:CT11F9 immune complex. The red circle shows that three CT11F9 molecules were bound near the viral capsid’s three-fold axis; (**B**) atomic model of the EV71:CT11F9 immune complex; (**C**,**D**) interaction interfaces between antibody CT11F9 heavy (**C**) and light (**D**) chains and EV71 particles. Green dashed lines show hydrogen bonds, and yellow dashed lines indicate salt bridges.

In the IVRP assay, MAb activity and epitope recognition are crucial. Studies confirm that CT11F9, targeting conformational epitopes, has broad-spectrum neutralizing activity against EV71 and can treat mice infected with EV71 [34,35]. To clarify the epitope recognized by CT11F9, high-purity EV71 particles were incubated with the Fab fragment of CT11F9. Cryo-EM was used to determine the electron density map of the EV71:CT11F9 complex at 3.04 Å resolution (Figure 1A, Table S1). Results show that EV71 particles maintain icosahedral symmetry after binding to CT11F9. The capsid was composed of four structural proteins (VP1, VP2, VP3, and VP4), forming a protomer. VP1, VP2, and VP3 were on the surface, while VP4 was internal. Sixty protomers formed a complete icosahedral virus particle, with 60 CT11F9 molecules bound per particle. Three CT11F9 molecules were bound near the viral capsid’s three-fold axis with C3 symmetry (Figure 1A,B).

To analyze the antigenic epitope where EV71 particles bind to CT11F9, an atomic model of the complex was built using the electron density map. Results showed that the interaction interface of the EV71:CT11F9 immune complex was mainly composed of the C-terminus of VP1, the BC and EF loops of VP2, and the AB loop of VP3, along with the CDR1-3 regions of the heavy chain and CDR1 and CDR3 of the light chain of CT11F9 (Figure 1C,D). Besides van der Waals forces, there were 17 hydrogen bonds and 4 salt bridges. Hydrogen bonds included VP1 T292-T30H, VP2 S74-R30L, S74-H91L, E159-R30L, VP3 N56-Y54H, T60 S31H, N61-Y33H, N61-D105H, A62-D105H, T63-Y101H, T63-D105H, E67-Y34H, and E67-Y54H. Salt bridges included VP2 E72-H91L and E159-R30L (Table S2). These results indicated that the neutralizing antibody CT11F9 recognizes a conformational epitope spanning VP1-3 of the same protomer, which can be used for establishing the IVRP.

##### Risk Assessment

Based on historical ELISA development experiences and data, brainstorming was used to draw an Ishikawa diagram (Figure S1) of influencing factors. The factors were ranked, and a failure mode and effect analysis (FMEA) table (Table S3) was made. Considering the impact of each factor on the accuracy, precision, and specificity defined in the ATP, the degree of impact was divided into five levels (1–5). Risks of influencing factors were scored based on these standards. Factors with scores over 70 were selected for the DoE design to optimize the method.

##### Method Optimization

Experiments were conducted based on the JMP DoE. The S/N ratio and positive control absorbance (S) were input into JMP for the Monte Carlo simulation (Figure S2). Parameters were set as follows: coating antibody incubation at 2–8 °C, diluted 1500–2500 times, for 17–28 h; sample incubation at 37 °C for 70–100 min; enzyme-labeled antibody incubation at 37 °C, diluted 6000–8000 times, for 90–100 min; color development at 37 °C for 8–15 min. After 10,000 Monte Carlo simulations, the experimental error probability was 0. These parameters were set as the method optimization and robustness testing (MODR). The optimal method parameters within the MODR were as follows: coating antibody diluted 2000 times, incubated overnight at 2–8 °C; samples incubated at 37 °C for 80 min; enzyme-labeled antibody diluted 7500 times, incubated at 37 °C for 100 min; color development at 37 °C for 10 min.

##### Model Selection

After determining the optimal method parameters, the standard curve of serially diluted standards was fitted using various models: the four-parameter logistic Rodbard (neither X nor Y ln-transformed), four-parameter logistic (neither X nor Y ln-transformed; X ln-transformed, Y not), and linear (X ln-transformed, Y not). As shown in Table S4, the four-parameter logistic Rodbard model (neither X nor Y ln-transformed) had the lowest AICc value and a growth rate close to 1.0, meeting the usage requirements. This model is commonly available in most enzyme-labeling instruments or analysis software, facilitating subsequent operations.

#### 3.2.2. Validation of the IVRP

##### Specificity

**Figure 2:**
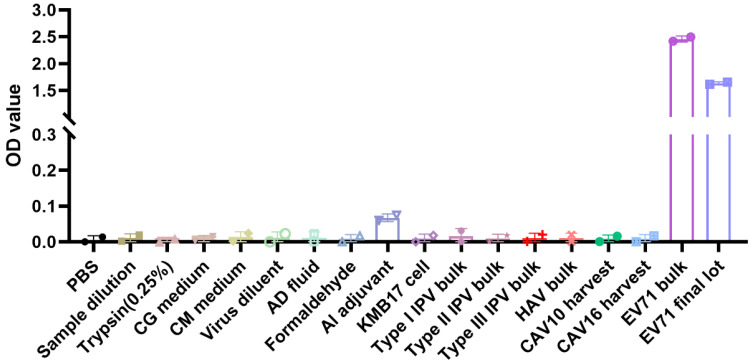
The specificity of the ELISA method. CG medium: cell growth medium; CM medium: cell maintenance medium; AD fluid: antigenic dissociation fluid; Al adjuvant: aluminum adjuvant; IPV bulk: Inactivated polio vaccine bulk; HAV bulk: Hepatitis A vaccine bulk; CAV10 harvest: Coxsackievirus group A type 10 harvest fluid; CAV16 harvest: Coxsackievirus group A type 16 harvest fluid.

Process-related impurities (PBS, trypsin, cell growth medium, virus diluent, formaldehyde) and other vaccines (inactivated polio vaccine (I, II, III) bulk, hepatitis A vaccine bulk, coxsackievirus group A type 10 and type 16 harvest fluid) from Manufacturer A were tested. Except for the EV71 bulk and final lot (OD > 1.5), all other samples had an OD < 0.1 (Figure 2), indicating that the method’s specificity meets the requirements.

##### Accuracy and Precision

Based on the working standard, samples with known relative potencies of 0.5, 0.71, 1.0, 1.41, and 2.0 were prepared. The relative potency of each sample was determined using the established IVRP method. The results were compared with the known relative potency to assess the method’s accuracy and precision. The results showed that the relative bias and 90% confidence interval of the measured relative potency at each level were within ±5%, and the intermediate precision was less than 7% (Table S5). Thus, the accuracy and precision of the newly established method met the preset ATP requirements.

##### Method Capability Evaluation

The overall capability, accuracy, and precision of IVRP were evaluated. Analysis of the measured values at each potency level showed that the 90% prediction intervals and tolerance intervals for each relative potency value were within the range of 70–141%. The total method error (method variability) for each relative potency value was ≤9%, the method misjudgment probability was <1%, and the method capability indices (MCIs) were grade II or higher (Table S6). For relative potency values ranging from 0.5 to 2.0, the established IVRP meets the expected objectives.

Based on neutralizing MAbs against a conformational epitope or epitopes (Table S7, Figure S3), the three manufacturers established IVRP methods for EV71 inactivated vaccines. Method validation showed that the specificity of each IVRP method was good. In the 0.5–2.0 relative potency range, the relative bias was ≤15%, intermediate precision was ≤15%, and total analytical error was ≤15%, meeting the preset ATP requirements (Table 2). This indicates that the IVRP methods established by the manufacturers are accurate and reliable for the quality control of EV71 inactivated vaccines.

### 3.3. Evaluated the IVRP Methods with Different Concentrations and Heat-Treated Vaccines

The WHO guidelines and the European Pharmacopoeia recommend that in vitro methods should initially be evaluated with samples at various concentrations. Subsequently, samples subjected to different stress conditions should be tested to evaluate the stability-indicating potential of the new method [7,9].

#### 3.3.1. Testing of EV71 Vaccines with Different Antigen Concentrations

**Figure 3:**
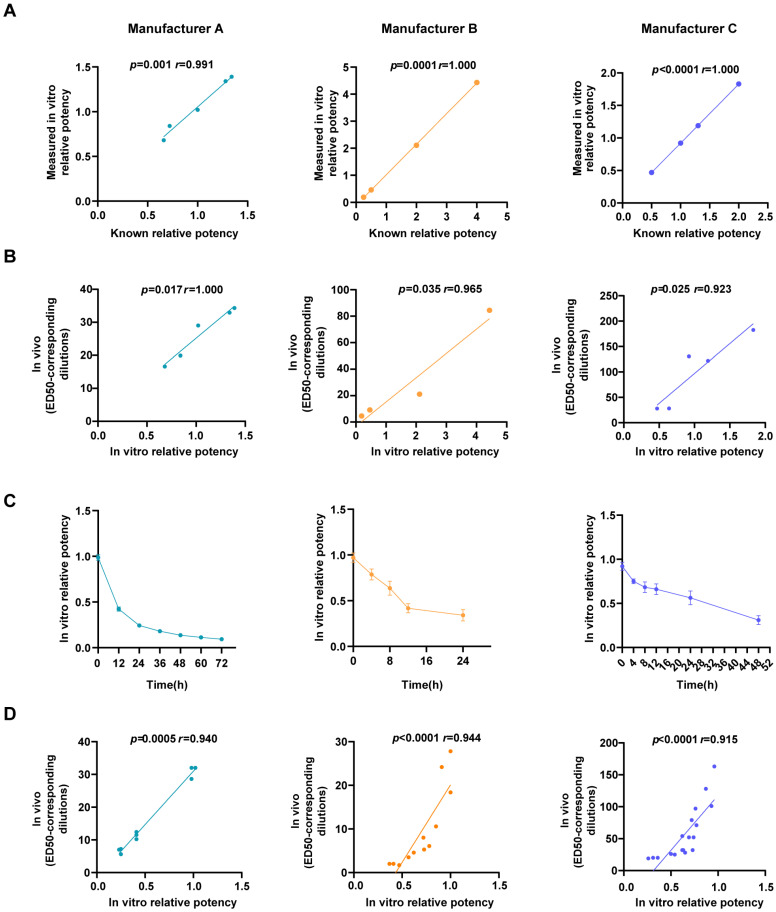
Correlation between measured IVRP values and known IVRP and between measured IVRP values and ED50-corresponding dilutions. (**A**) Correlation between known and measured IVRP values; (**B**) correlation between IVRP values and ED50-corresponding dilutions; (**C**) IVRP values of 56 °C heat-treated samples; (**D**) correlation between IVRP values and ED50-corresponding dilutions of heat-treated samples. Spearman correlation analysis was used, with *p* < 0.05 indicating statistical significance and r > 0.8 indicating a strong correlation.

EV71 vaccines with different antigen concentrations were tested using the IVRP and in vivo method (ED50). The results indicated that the in vitro method could differentiate between different antigen concentrations (*p* < 0.01) (Figure 3A). The measured values were highly consistent with known values (r > 0.99). Significant correlations were identified between IVRP values and ED50-corresponding dilutions (*p* < 0.05, r > 0.9) (Figure 3B). These results suggested that the in vitro method can reflect differences in antigen concentrations and detect process variations.

#### 3.3.2. Testing of Heat-Treated Samples

EV71 vaccines were incubated at 56 °C for different amounts of time and then measured by IVRP and in vivo method. The values of IVRP decreased as the incubation at 56 °C was prolonged (Figure 3C). For heat-treated vaccines, the values of IVRP were significantly correlated with the dilutions corresponding to ED50 (*p* < 0.001, r > 0.9) (Figure 3D). These results indicated that IVRP can reflect the change in stability.

### 3.4. Establishment of Potency Specifications

The establishment of specifications is crucial for the research and development of biological products. The determination of vaccine potency specifications is associated with vaccine stability, total analytical error of the method, variations in the manufacturing process, and the data obtained for lots used in clinical studies [36]. In this study, a working standard with immunogenicity traceable to the phase 3 clinical vaccine and stability equivalent to that of the tested vaccine was used. Additionally, the IVRP methods were used to present the activity of each commercial product. Therefore, the IVRP specifications of EV71 vaccines can ignore the impact of vaccine stability, only consider the IVRP values of multiple batches of commercial vaccines, and the total error of the IVRP methods (all below 10%).

**Figure 4:**
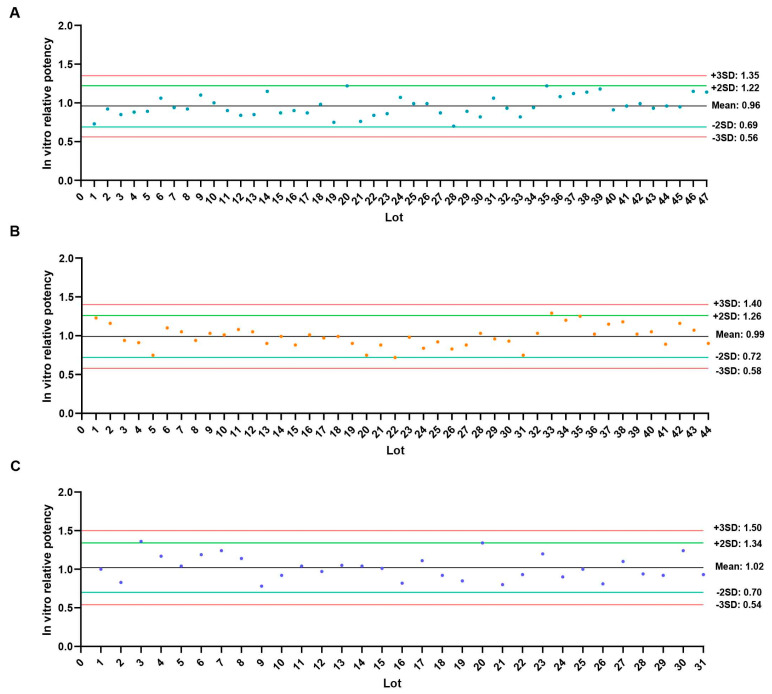
IVRP values of multiple batches of commercial vaccines from different manufacturers. (**A**). Manufacturer A; (**B**). Manufacturer B; (**C**). Manufacturer C.

The ED50 values of all commercial vaccines met the current in vivo potency specifications. Using the GraphPad software to plot individual control charts of IVRP results: Manufacturer A’s 47 batches of the EV71 final lot had a maximum IVRP value of 1.22, a minimum of 0.70, and a mean ±3 SD range of 0.56–1.35; Manufacturer B’s 44 batches had a maximum IVRP value of 1.29, a minimum of 0.72, and a range of 0.58–1.40; Manufacturer C’s 31 batches had a maximum IVRP value of 1.36, a minimum of 0.78, and a range of 0.54–1.50 (Figure 4A–C). Thus, the IVRP specifications of the EV71 vaccines of each company were set at 0.56–1.35, 0.58–1.40, and 0.54–1.50, respectively.

Analysis of samples treated at 56 °C using the proposed IVRP specifications showed that heat-treated samples from Manufacturers A, B, and C at 12 h, 8 h, and 24 h fell below the specifications (Tables S8–S10). When tested with the in vivo method, the 12 h heat-treated samples from Manufacturer B exceeded the current specification (Manufacturer B ≤ 100 U), while Manufacturer C’s 48 h heat-treated sample remained within the specification (≤100 U) (Tables S9 and S10). This indicates that the new IVRP methods were more sensitive than the in vivo method in detecting antigen activity changes.

## 4. Discussion

Over 8.05 million animals were used for research and testing in the EU in 2020, with 17% used for regulatory purposes [37]. With the widely acknowledged 3R (Replacement, Reduction, Refinement) principle, the WHO, regulatory authorities, and vaccine manufacturers are all engaged in developing alternative methods for the batch testing of vaccines [7,38,39,40,41]. However, only a few vaccines have completed the substitution of potency tests. Alternative research is ongoing for rabies, diphtheria, pertussis, and other vaccines [11]. For rabies vaccines, the European Partnership for Alternatives to Animal Testing (EPAA) has conducted studies with multiple laboratories. The results show that methods based on two neutralizing MAbs (D1-25, 1112-1) targeting the rabies G protein can distinguish changes in antigen content of inactivated rabies vaccines. This method may provide a unified method for replacing the NIH method [13]. However, Volokhov et al. found that recombinant rabies glycoprotein expressed by plant expression systems could not induce neutralizing antibodies, but the IVRP method based on D1-25 and 1112-1 could detect antigen content [42]. This suggests that a single method may not meet the testing needs of vaccines produced by multiple processes. Moreover, for the same vaccines produced by different manufacturers with different strains and processes, developing new testing methods based on product characteristics may lead to issues regarding the consistency and compliance of method replacement. Additionally, the large variability in in vivo methods makes them challenging to correlate with in vitro methods. In addition, the application of in vitro methods alters the specification, increasing the complexity of approval.

To guide the development and implementation of in vitro methods as substitutes for existing in vivo methods, the European Pharmacopoeia added a general chapter in 2018, and the WHO published a draft guideline in 2024 [7,9]. These guidelines describe the advantages of in vitro methods, clarify the prerequisites for method replacement, and propose specific suggestions for method performance and validation. They also indicate that establishing a unified test method for a class of products is challenging and should not be a prerequisite for method replacement.

The EV71 inactivated vaccines were developed by NIFDC and three manufacturers (Kunming Institute, Wuhan Institute, and Sinovac) to address the serious public health issue caused by HFMD [2]. To solve the key technical bottlenecks in antigen quantification and immunogenicity evaluation, the NIFDC conducted the establishment of antigen content and neutralizing antibody standards at the initial research of EV71 vaccines [43]. The national standards provided benchmarks for vaccines produced by different strains, cell substrates, and processes, achieving uniform production and specification [43]. Additionally, the relevant research provides the basis for the development of WHO international standards and the development of guidelines [44,45]. Nine years after the EV71 inactivated vaccines were launched, collaborative research on in vitro alternative methods was organized by NIFDC, supported by the Chinese Pharmacopoeia Commission, and performed by three manufacturers. The WHO guidelines and the European Pharmacopoeia were referred to. The main objective of this study was to establish working standards, standardized IVRP methods, and IVRP specifications for manufacturers with differences in strains and production processes.

The three EV71 vaccine manufacturers used different C4-type EV71 strains as vaccine strains, with differences in cell lines (two used Vero cells and one used human diploid cells) and processes [2]. These differences affected vaccine dissociation and immunogenicity. Previous research showed that variations in vaccine dissociation could lead to a noncommutability of unified standards. Thus, inaccurate quantification was generated. To avoid this, each manufacturer used commercial vaccines with immunogenicity similar to phase 3 clinical vaccines as working standards, following WHO guidelines and the Chinese Pharmacopoeia [21,22]. To address the issue of comparability in quantitative results among three manufacturers, we calibrated each manufacturer’s working standards using national standards that trace to the EV71 International standard. The uncertainty of these working standards was determined by calculating the 95% confidence interval of the calibration values.

In developing the IVRP method, MAb selection was crucial. Each company conducted antibody screening or epitope research based on its vaccine strain. CT11F9 MAb possessed broad-spectrum neutralizing activity against EV71 subtypes 1 and 2.1–2.4 and had a good treatment effect in mice infected with EV71 [34,35]. The epitopes recognized by CT11F9 were immunodominant in humans. In our study, Cryo-EM studies were conducted to characterize the epitopes CT11F9 recognized. We found that CT11F9 recognized epitopes spanning VP1-3 of the same protomer. This suggested that an IVRP method based on CT11F9 could align in vitro and in vivo test results. So, Manufacturer A used CT11F9 to establish an IVRT method. The MAbs used by the other two manufacturers also had good neutralizing activity and targeted conformational epitopes on the viral capsid.

The development and validation of biological activity assays have been updated recently. USP 1220 and ICH Q14/Q2 (R2) provide a framework for the implementation of the life cycle approach [18,19,46]. The approach includes the identification of critical quality attributes, the establishment of an ATP, the identification of analytic procedure parameters, the optimization of parameters, the validation, and the ongoing monitoring method. The draft of the 2025 Chinese Pharmacopoeia also incorporates these concepts [20]. An enhanced approach facilitates the comprehension of method variability, enhances the handling of out-of-specification (OOS) results, and supports the continual improvement of the method throughout its lifecycle [18]. Previously, we used this approach to develop and validate methods for SARS-CoV-2 protein subunit vaccine antigen quantification and SARS-CoV-2 antibody analysis [47,48]. The three EV71 vaccine manufacturers also applied this approach to establish and validate IVRP methods. During IVRP method development, accuracy and precision were limited to within 15% and total analytical error to below 20%. Critical factors like coating and detection antibody ratios and colorimetric time were optimized using single- or multi-factor DoE. Multi-person repeated tests confirmed that the new methods met preset ATP requirements, with good accuracy and precision, effectively meeting EV71 inactivated vaccine testing needs. Each manufacturer prepared EV71 vaccines with different antigen concentrations and heat-treated samples to evaluate the ability to distinguish sub-potent samples. The results showed that the IVRP methods could distinguish the changes in antigen content and had a good correlation with ED50 dilutions, suggesting they could replace in vivo methods.

Vaccine potency specification should be developed by considering product stability, the total analytical error of the method, process variations across batches, and clinical data [36]. The total error of the established EV71 IVRP method was less than 10%. In addition, the IVRP methods used working standards with immunogenicity traceable to phase 3 clinical vaccines. Therefore, potency specifications were set based on multiple commercial batches. Using the potency specification to evaluate the samples subjected to 56 °C, it was observed that the IVRP methods employed by Manufacturers B and C could detect failure samples with greater sensitivity than the in vivo methods. For Manufacturer A, the consistency in determining the failure samples between in vitro and in vivo methods may be attributed to the absence of adequately prepared heat-treated samples.

## 5. Conclusions

According to WHO guidelines and the European Pharmacopoeia, the NIFDC and three Chinese EV71 inactivated vaccine manufacturers established sensitive IVRP methods for detecting process variations. Additionally, the potency specifications of IVRP methods were set. IVRP methods have the potential to replace the currently used in vivo method and can serve as rapid methods for emergency testing.

## Figures and Tables

**Table 1:** Information on working standards of each manufacturer.

		A	B	C
Comparison of working standard (WS) and Phase 3 vaccine (P3V)	The ratio of ED50 (WS/P3V)	1.49	0.90	2.04
	Antigen content ratio after dissociation (WS/P3V)	1.25	1.20	0.91
EV71 antigen content of WS (IU/mL)		242 (95% CI 240–245)	812 (95% CI 793–830)	999 (95% CI 985–1012)

**Table 2:** ATP and comparison with final development results.

	Objectives	Results		
		Manufacturer A	Manufacturer B	Manufacturer C
Intended purpose	Establishing an in vitro relative potency method that can be used for batch testing and stability testing of the final lot of EV71 inactivated vaccine.			
Critical quality attribute (CQA) and experimental principle	CQA: The antigen content of EV71 inactivated vaccines. Experimental principle: The EV71 polyclonal antibody was immobilized on the solid phase. An immune complex with the antigen in the sample would form. After washing, an enzyme-labeled MAb targeting conformational epitopes relevant to the protection offered by the vaccines was added. Thereby, an enzyme-labeled antibody–antigen–antibody complex–solid phase complex was formed. After subsequent washing, substrate was added for color development. The optical density in the microplate wells was directly proportional to the concentration of the analyte. The in vitro relative potency of the sample compared to the standard product is then calculated.			
Specificity	No cross reaction with hepatitis A virus, influenza virus, and other enteroviruses			
Accuracy (relative bias)	The relative bias of each known relative potency should be less than 15%, and its 90% confidence interval should be in the range of ±20%	In the known relative potency range of 0.5–2.0, the relative bias of each level was less than 2%, and the 90% confidence limit was not higher than ±5%	In the known relative potency range of 0.5–2.0, the relative bias of each level was less than 10%, and the 90% confidence limit was not higher than ±15%	In the known relative potency range of 0.5–2.0, the relative bias of each level was less than 10%, and the 90% confidence limit was not higher than ±15%
Intermediate precision	≤15%	≤9%	≤8%	≤8%
Total analytical error	≤20%	≤9%	≤10%	≤8%

## Data Availability

The cryo-EM density maps and corresponding atomic coordinates have been deposited in the Electron Microscopy Data Bank (EMDB) (https://www.ebi.ac.uk/emdb/ (accessed on 11 March 2025)) and Protein Data Bank (PDB) (https://www.rcsb.org (accessed on 11 March 2025)), respectively. The accession code is EV71:CT11F9 (EMD-63694, PDB:9M7V). Other data generated or analyzed during this study are available from the corresponding authors upon reasonable request.

## Supplementary Materials

The following supporting information can be downloaded at https://www.mdpi.com/article/10.3390/vaccines13040404/s1, Table S1. Cryo-EM data collection, refinement, and validation statistics of EV71:CT11F9; Table S2. Summary of hydrogen and salt bond interactions between EV71 particles and CT11F9 MAb; Table S3. Failure mode effect analysis (FMEA); Table S4. Model parameter of the IVPR method developed by Manufacturer A; Table S5. Analysis of accuracy and precision results (n = 17); Table S6. Evaluation of method capability at different relative potency levels; Table S7. Information on MAbs; Table S8. IVRP and ED50 results of 56 °C heat-treated samples of Manufacturer A; Table S9. IVRP and ED50 results of 56 °C heat-treated samples of Manufacturer B; Table S10. IVRP and ED50 results of 56 °C heat-treated samples of Manufacturer C; Figure S1. Factors that may affect the results of in vitro relative potency tests are summarized in an Ishikawa diagram; Figure S2. Screening for optimal ELISA experimental conditions; Figure S3. Characterization of EV71-specific MAbs.
