# Supplementary material for: Development of In Vitro Potency Methods to Replace In Vivo Tests for Enterovirus 71 Inactivated Vaccine (Human Diploid Cell-Based/Vero Cell-Based)
Source: Vaccines (Basel). 2025 Apr 13;13(4):404. doi: 10.3390/vaccines13040404 (PMC12031293; doi:10.3390/vaccines13040404)
Supplement: Supplementary file 1 [file vaccines-13-00404-s001.zip › vaccines-3550846-supplementary.pdf]

Table S1. Cryo-EM data collection, refinement and validation statistics of EV71:CT11F9

**Data collection and processing**	
Microscope	TF30
Detector	K3
Magnification	31,000
Voltage (kV)	300
Electron exposure (e-/Å^2)	40
Defocus range (μm)	0.7-2.9
Pixel size (Å)	0.5
Symmetry imposed	I2
Micrographs (no.)	3,820
Final particle images (no.)	71,798
Map resolution (Å)	3.04
FSC threshold	0.143
Map sharpening B factor (Å^2)	-166.3
**Validation**	
MolProbity score	1.05
Clashscore	1.77
Poor rotamers (%)	0
RMS (bonds)	0.003
RMS (angles)	0.52
**Ramachadran plot**	
Favored (%)	97.4
Allowed (%)	2.6
Disallowed (%)	0

Table S2. Summary of hydrogen and salt bond interactions between EV71 particles and CT11F9 MAb

Mature virion	Domains	Residus	Distance (Å)	CT11F9-Fab	CDR	Interaction
VP1	C-termini	T 292[OG1]	3.65	T 30[OG1]	H-CDR1	Hydrogen bond
VP2	BC loop	E 72[OE2]	3.67	H 91[NE2]	L-CDR3	Salt bridge
		S 74[O]	3.16	R 30[NH2]	L-CDR1	Hydrogen bond
		S 74[OG]	3.21	H 91[O]	L-CDR3	Hydrogen bond
	EF loop	E 159[OE2]	3.73	R 30[NE]	L-CDR1	Hydrogen bond and salt bridge
		E 159[OE1]	3.78	R 30[NH2]	L-CDR1	Hydrogen bond and salt bridge
		E 159[OE1]	3.91	R 30[NE]	L-CDR1	Salt bridge
VP3	AB loop	N 56[ND2]	3.88	Y 54[OH]	H-CDR2	Hydrogen bond
		T 60[N]	3.26	S 31[O]	H-CDR1	Hydrogen bond
		T 60[OG1]	3.48	S 31[O]	H-CDR1	Hydrogen bond
		N 61[ND2]	2.65	D 105[OD2]	H-CDR3	Hydrogen bond
		N 61[OD1]	3.19	Y 33[N]	H-CDR1	Hydrogen bond
		A 62[N]	3.58	D 105[OD2]	H-CDR3	Hydrogen bond
		T 63[OG1]	3.33	Y 101[N]	H-CDR3	Hydrogen bond
		T 63[N]	3.31	D 105[OD2]	H-CDR3	Hydrogen bond
		T 63[OG1]	2.82	D 105[OD2]	H-CDR3	Hydrogen bond
		E 67[OE1]	3.25	Y 34[OH]	H-CDR1	Hydrogen bond
		E 67[OE2]	3.06	Y 34[OH]	H-CDR1	Hydrogen bond
		E 67[OE2]	3.41	Y 54[OH]	H-CDR2	Hydrogen bond

The interaction residues (distances cut-off: 4.0) were computed using PISA server (https://www.ebi.ac.uk/pdbe/pisa/).

Table S3. Failure mode effect analysis（FMEA）

Rating of importance			8	8	10	
ID	process step	process input	Accuracy	Specificity	Precision	Total
1	Environment	Temperature	1	1	1	26
2	Environment	Humidness	1	1	1	26
3	Microplate reader	Brand	1	1	1	26
4	Calculation	Software	1	1	1	26
5	Substrate	Volume	2	1	2	44
6	Microplate	Brand	2	1	2	44
7	Substrate	Brand	2	1	2	44
8	Analyst	Training	2	1	2	44
9	Analyst	Practice time	2	1	2	44
10	Consumable	Absorbance	2	1	2	44
11	Washing plate	Volume	2	1	2	44
12	Coating	Volume	2	1	2	44
13	Blocking	Time	2	2	2	52
14	Blocking	Temperature	2	2	2	52
15	Coating	Temperature	2	1	3	54
16	Coating buffer	Formula	2	3	3	70
17	Substrate	Temperature	3	1	4	70
18	Washing buffer	Formula	3	2	3	70
19	Sample dilution buffer	Formula	3	1	4	72
20	Dilution buffer of enzyme labeled MAb	Formula	3	1	4	72
21	Coating buffer	Formula	2	1	4	72
22	Test sample	Time	3	1	4	72
23	Test sample	Temperature	3	1	4	72
24	Enzyme labeled MAb	Concentration	3	1	4	72
25	Enzyme labeled MAb	Time	3	1	4	72
26	Enzyme labeled MAb	Temperature	3	1	4	72
27	Coating	Concentration of polyclonal antibody	4	1	4	80
28	Coating	Time	4	1	4	80
29	Washing plate	Times	2	3	4	80
30	Calculation	Concentration point setting	4	1	4	80
31	Analyst	Ability	4	1	4	80
32	Calculation	Model	4	1	4	80

33	Test sample	Concentration	3	2	4	80
34	Microplate washer	Maintenance	4	3	4	96
35	Enzyme labeled MAb	Targeted epitopes	4	3	4	96
36	Substrate	Time	4	3	5	106

Table S4. Model parameter of the IVPR method developed by Manufacturer A

**Model**	**AICc**	**Growth rate/slope**	**R^2**
Four-parameter logistic Rodbard	-359.995	1.058	1.000
Four-parameter logistic	-105.623	9.632	0.997
Four-parameter logistic (ln(x), y)	-287.850	1.048	1.000
Linear (ln(x), y)	-121.950	0.418	0.994

Table S5. Analysis of accuracy and precision results (n=17)

	Know relative potency				
	0.5	0.71	1.0	1.41	2.0
Relative bias (%)	-0.416	-0.701	1.704	0.386	-0.951
90% confidence interval of relative bias (%)	-2.441~1.651	-3.345~-2.016	-0.300~3.748	-1.597~-2.410	-3.400~-1.561
Intermediate precision (%)	8.128	8.312	6.283	6.276	7.675

Table S6. Evaluation of method capability at different relative potency levels

Evaluation of method capability	Known relative potency levels				
	0.5	0.71	1.0	1.41	2.0
Method variability (%)	8.140	8.345	6.527	6.289	7.741
MCI	1.461	1.427	1.808	1.875	1.534
Misjudgment probability	1.176E-05	1.888E-05	4.307E-08	1.826E-08	4.257E-06
90% Prediction interval (%)	86.52-114.62	85.98-114.68	90.78-113.94	89.97-112.01	86.63-113.25
90% Tolerance interval (%)	83.71-118.46	83.12-118.62	88.39-117.02	87.68-114.63	83.95-116.87
Method level	II	II	I	I	II

Table S7. Information on MAbs

Number	Name	Manufacturer	Conformational or linear epitopes	Activity	Target epitopes
1	CT11F9	A	Conformational	Neutralizing	VP1~3
2	8A4	B	Conformational	Neutralizing	VP2,VP3
3	H07-080902	C	Conformational	Neutralizing	VP1

Table S8. IVRP and ED50 results of 56℃ heat-treated samples of Manufacturer A

Incubation time at 56°C	IVRP			ED50（U）		
	Sample1	Sample2	Sample3	Sample1	Sample2	Sample3
0h	1.02	0.98	0.96	7.8	7.8	8.7
12h	**0.41**	**0.41**	**0.45**	**24.5**	**20.2**	**21.7**
24h	**0.23**	**0.25**	**0.25**	**35.7**	**44.6**	**34.7**

Note: Black is the value in the potency specifications (IVRP: 0.56-1.35, *in vivo* potency: ≤15 U); Red marks are values that exceed the potency specifications.

Table S9. IVRP and ED50 results of 56℃ heat-treated samples of Manufacturer B

Incubation time at 56°C	IVRP			ED50（U）		
	Sample1	Sample2	Sample3	Sample1	Sample2	Sample3
0h	1.00	1.00	0.91	11.5	17.4	13.2
4h	0.78	0.85	0.73	52.8	30.3	60.6
8h	0.62	0.72	**0.57**	69.6	40	91.9
12h	**0.41**	**0.47**	**0.37**	**160**	**183.8**	**160**
24h	**0.36**	**0.39**	**0.27**	N/A	N/A	N/A

Note: Black is the value in the potency specifications (IVRP: 0.58-1.40, *in vivo* potency: ≤100 U); Red marks are values that exceed the potency specifications. N/A: not available.

Table S10 IVRP and ED50 results of 56℃ heat-treated samples of Manufacturer C

Incubation time at 56°C	IVRP			ED50（U）		
	Sample1	Sample2	Sample3	Sample1	Sample2	Sample 3
0h	0.96	0.87	0.93	6	8	10
4h	0.76	0.72	0.77	10	12	14
8h	0.74	0.62	0.69	18	18	18
12h	0.73	0.63	0.62	30	30	30
24h	0.65	**0.5**	0.54	34	37	38
48h	**0.36**	**0.26**	**0.31**	47	49	47

Note: Black is the value in the potency specifications (IVRP: 0.54-1.50, *in vivo* potency: ≤100 U); Red marks are values that exceed the potency specifications.

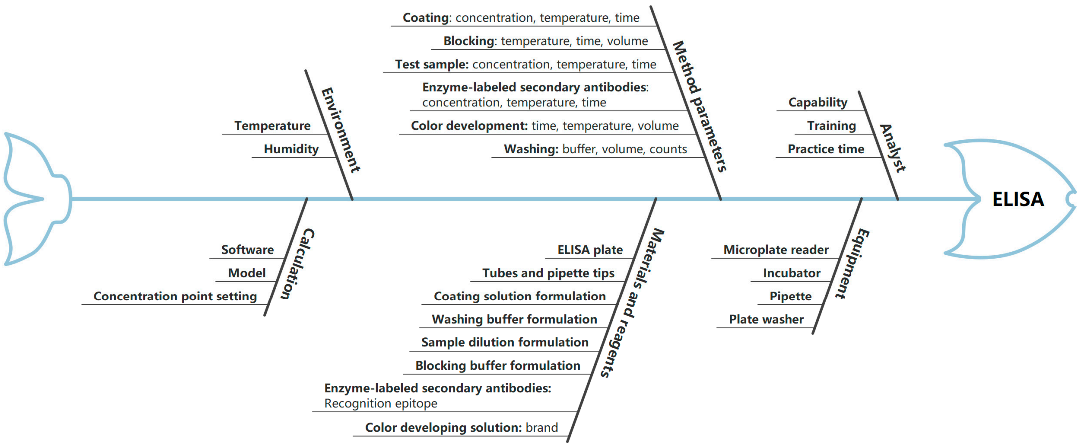

Figure S1. Factors that may affect the results of *in vitro* relative potency tests were summarized in the Ishikawa diagram. The factors were divided into six parts: analyst, equipment, method parameters, environment, calculation, materials, and reagents.

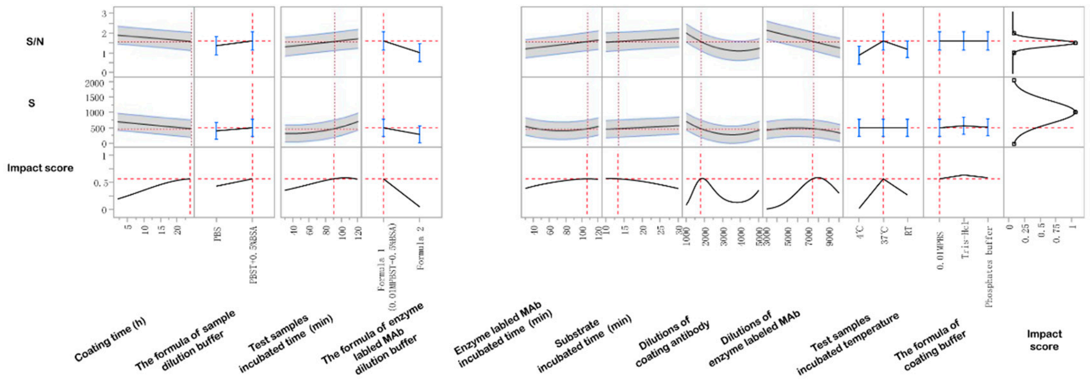

Figure S2. Screening for optimal ELISA experimental conditions. The dynamics of each influencing factor (i.e., coating time, the formula of sample dilution buffer) were visualized using Monte Carlo simulations. Impact score considers the response importance weight; the closer to 1, the better the impact score. S/N: signal-to-noise ratio. S: signal value.

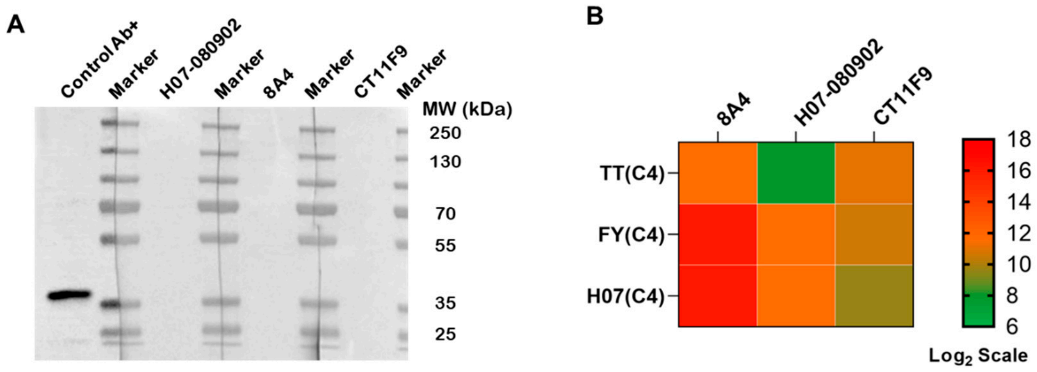

Figure S3. Characterization of EV71 specific MAbs. A: Western Blot detection of MAbs recognition epitope; B: Heat map representing the neutralizing activity of MAbs against viruses used in the production of EV71 vaccines. MAbs adjusted to 1 mg/mL were serially diluted 2-fold for 8 dilutions. Neutralizing titer was defined as the highest dilution of MAbs that inhibited 50% of the CPE after 7 days. The titer values were log2 transformed and were color coded as indicated by the log2 scale on the right, in which signal intensities range from red to green, indicating high and low titers, respectively. Control Ab + was an MAb specific for linear epitopes. CT11F9, 8A4, and H07-080902 were conformational monoclonal antibodies screened by Companies A, B, and C, respectively. FY(C4), TT(C4), and H07(C4) were vaccine strains used by Manufacturers A, B, and C.
